# Comprehensive Antiretroviral Restriction Factor Profiling Reveals the Evolutionary Imprint of the *ex Vivo* and *in Vivo* IFN-β Response in HTLV-1-Associated Neuroinflammation

**DOI:** 10.3389/fmicb.2018.00985

**Published:** 2018-05-22

**Authors:** Fabio E. Leal, Soraya Maria Menezes, Emanuela A. S. Costa, Phillip M. Brailey, Lucio Gama, Aluisio C. Segurado, Esper G. Kallas, Douglas F. Nixon, Tim Dierckx, Ricardo Khouri, Jurgen Vercauteren, Bernardo Galvão-Castro, Rui Andre Saraiva Raposo, Johan Van Weyenbergh

**Affiliations:** ^1^Oncovirology Program, Instituto Nacional de Câncer (INCA), Rio de Janeiro, Brazil; ^2^Microbiology Immunology and Tropical Medicine, George Washington University, Washington, DC, United States; ^3^Department of Microbiology and Immunology, Rega Institute for Medical Research, KU Leuven, Leuven, Belgium; ^4^Departamento de Moléstias Infecciosas e Parasitárias, Faculdade de Medicina, Universidade de São Paulo, São Paulo, Brazil; ^5^Department of Molecular and Comparative Pathobiology, Johns Hopkins University School of Medicine, Baltimore, MD, United States; ^6^Fundação Oswaldo Cruz, Instituto Gonçalo Moniz (IGM), Salvador-Bahia, Brazil; ^7^Escola Bahiana de Medicina e Saúde Pública, Salvador-Bahia, Brazil

**Keywords:** HTLV-1, HIV, retrovirus, evolution, interferon, neuroinflammation, multiple sclerosis, transcriptomics

## Abstract

HTLV-1-Associated Myelopathy (HAM/TSP) is a progressive neuroinflammatory disorder for which no disease-modifying treatment exists. Modest clinical benefit from type I interferons (IFN-α/β) in HAM/TSP contrasts with its recently identified IFN-inducible gene signature. In addition, IFN-α treatment *in vivo* decreases proviral load and immune activation in HAM/TSP, whereas IFN-β therapy decreases tax mRNA and lymphoproliferation. We hypothesize this “IFN paradox” in HAM/TSP might be explained by both cell type- and gene-specific effects of type I IFN in HTLV-1-associated pathogenesis. Therefore, we analyzed *ex vivo* transcriptomes of CD4^+^ T cells, PBMCs and whole blood in healthy controls, HTLV-1-infected individuals, and HAM/TSP patients. First, we used a targeted approach, simultaneously quantifying HTLV-1 mRNA (HBZ, Tax), proviral load and 42 host genes with known antiretroviral (anti-HIV) activity in purified CD4^+^ T cells. This revealed two major clusters (“antiviral/protective” vs. “proviral/deleterious”), as evidenced by significant negative (TRIM5/TRIM22/BST2) vs. positive correlation (ISG15/PAF1/CDKN1A) with HTLV-1 viral markers and clinical status. Surprisingly, we found a significant inversion of antiretroviral activity of host restriction factors, as evidenced by opposite correlation to *in vivo* HIV-1 vs. HTLV-1 RNA levels. The anti-HTLV-1 effect of antiviral cluster genes was significantly correlated to their adaptive chimp/human evolution score, for both Tax mRNA and PVL. Six genes of the proposed antiviral cluster underwent lentivirus-driven purifying selection during primate evolution (TRIM5/TRIM22/BST2/APOBEC3F-G-H), underscoring the cross-retroviral evolutionary imprint. Secondly, we examined the genome-wide type I IFN response in HAM/TSP patients, following short-term *ex vivo* culture of PBMCs with either IFN-α or IFN-β. Microarray analysis evidenced 12 antiretroviral genes (including TRIM5α/TRIM22/BST2) were significantly up-regulated by IFN-β, but not IFN-α, in HAM/TSP. This was paralleled by a significant decrease in lymphoproliferation by IFN-β, but not IFN-α treatment. Finally, using published *ex vivo* whole blood transcriptomic data of independent cohorts, we validated the significant positive correlation between TRIM5, TRIM22, and BST2 in HTLV-1-infected individuals and HAM/TSP patients, which was independent of the HAM/TSP disease signature. In conclusion, our results provide *ex vivo* mechanistic evidence for the observed immunovirological effect of *in vivo* IFN-β treatment in HAM/TSP, reconcile an apparent IFN paradox in HTLV-1 research and identify biomarkers/targets for a precision medicine approach.

## Background

Human T-cell Lymphotropic Virus-1 (HTLV-1), recently renamed Human T-cell Leukemia Virus-1 due to its strong oncogenic potential (Gallo et al., 2017; Tagaya and Gallo, 2017), is also the causative agent of the debilitating neuroinflammatory disorder, HTLV-1-Associated Myelopathy/Tropical Spastic Paraparesis (HAM/TSP) (Osame et al., 1986). HAM/TSP is associated with high HTLV-1 proviral load (PVL) and transcriptional levels of retroviral regulatory genes, *Tax* and *HBZ* (Saito et al., 2009). The pathogenesis of HAM/TSP is complex (Bangham et al., 2015), and a proportion of HTLV-1 asymptomatic carriers (AC) may present a PVL and inflammatory profile similar to HAM/TSP patients but do not develop clinical symptoms. It is unknown why 2–3% of HTLV-1 infected patients develop HAM/TSP after years of latent infection, but an interferon-inducible gene signature has been identified in HAM/TSP and is absent in AC (Tattermusch et al., 2012). However, type I interferon-based clinical trials reported modest clinical benefit, as well as antiviral (decreased PVL, *Tax* mRNA levels) and immunomodulatory effects (decreased T-cell spontaneous proliferation and activation), suggesting IFN-α and IFN-β may hamper disease progression (Izumo et al., 1996; Oh et al., 2005). To reconcile this apparent contradiction, we hypothesize specific IFN-stimulated genes, e.g. antiviral effector genes, can exert deleterious vs. protective roles in HAM/TSP, as we recently suggested for B-cell expression of CD80 and CD86 (Menezes et al., 2014).

In contrast to HTLV-1, several anti(retro)viral effector genes have been classified as “restriction factors” (RFs) for HIV-1 infection, some of which are modulated by type I IFNs (Foster et al., 2017). We have previously compiled and validated a real-time PCR-array of 42 well-characterized RFs with suppressive activity against HIV-1 (Neil et al., 2008; Abdel-Mohsen et al., 2013; Raposo et al., 2013a,b, 2014), including APOBECs, TRIMs, and BST2/Tetherin. Enhanced HIV-1 infection upon siRNA-mediated silencing in neuroblasts illustrates the relevance of TRIM5α and TRIM22 in neuroinfection (Singh et al., 2014).

To assess the role of antiretroviral genes in HAM/TSP pathogenesis, we determined transcriptional levels of 42 RFs and regulatory HTLV-1 genes *Tax* and *HBZ* in CD4^+^ T cells from HTLV-1 patients. We identified a strong negative association between expression of HTLV-1 *Tax* and a cluster of RFs, including TRIM5α/TRIM22/BST2. Genome-wide transcriptomic analysis of HAM/TSP patients showed a significant and distinct up-regulation of RFs after *ex vivo* exposure to IFN-β, but not IFN-α. These results provide mechanistic evidence for the immunovirological impact of IFN-β therapy observed *in vivo* in HAM/TSP patients and pave the way to an evidence-based precision medicine approach to this neuroinflammatory disorder.

## Patients and methods

All participants signed a written informed consent in accordance with the Declaration of Helsinki, approved by the University Institutional Review Boards of USP (#0855/08) and CPqGM-FIOCRUZ (#022/03) before inclusion in the study. *Ex vivo* analysis of antiretroviral restriction factors was conducted in a cohort of 18 individuals (7 HAM/TSP, 6 HTLV-1-infected asymptomatic carriers (AC) and 5 age- and gender-matched HTLV-1-negative healthy controls (HC)), enrolled at the HTLV-1 Outpatient Clinic at the University of Sao Paulo (USP), Brazil. The second cohort (lymphoproliferation/microarray analysis) consisted of 10 HAM/TSP patients, followed at the Bahiana School of Medicine and Public Health HTLV reference center in Salvador-Bahia, Brazil. Validation of transcriptomic findings was performed in a third, published cohort (London, UK). Demographic and clinical data of all cohorts are detailed in Table 1. Clinical status was determined based on WHO criteria for HTLV-1 associated diseases (Osame, 1990). None of the HAM/TSP patients had received prior IFN-based therapy. Blood samples were obtained and processed with Ficoll-Paque PLUS (Amersham Pharmacia Biotech, Uppsala, Sweden) gradient centrifugation and peripheral-blood mononuclear cells (PBMC) were isolated and either cryopreserved in 10 % DMSO in FBS (RF analysis) or used for short-term *ex vivo* culture (48 h for microarray analysis, 96 h for lymphoproliferation), in the absence or presence of 1,000 IU/ml clinical grade IFN-α (gift of Blausiegel Ltda., Sao Paulo-Brazil) or IFN-β (gift of Dr. Brassat, Toulouse-France), as previously described (Moens et al., 2012a; Dierckx et al., 2017).

**Table 1:** Demographic and clinical data of 3 HTLV-1 cohorts.

**Cohorts**	**HC**	**AC**	**HAM/TSP**	**Cell type**
**Cohort 1 (Sao Paulo, BR)**				CD4+ T cells
Number of samples	5	6	7	
Age (median, IQR)	54(38–61)	62(54–66)	51(45–62)	
Gender	1M/4F	1M/5F	2M/5F	
PVL (median, ± SD)	N/A	7.6 ± 151.5	181.0 ± 134.5	
Tax mRNA (median, ± SD)	N/A	0.02 ± 0.02	0.68 ± 0.77	
HBZ mRNA (median, ± SD)	N/A	10^−5^ ± 5.4 10^−4^	2.7 10^−4^ ± 8.1 10^−4^	
**Cohort 2 (Bahia, BR)**				PBMC
Number of samples	11	5	10	
Age (median, IQR)			52 ± 6.2	
Gender	4M/7F	2M/3F	4M/6F	
**Cohort 3 (published; London, UK)**				Whole blood
Number of samples	8	17	10	
Age (median, IQR)	49(46–71)	47(38–62)	54(49–62)	63(44–74)
Gender	4M/4F	0M/17F	4M/6F	
PVL (median, IQR)	N/A	0.6(0.4–0.9)	3.9 (3.4–6.7)	10.1(6.7–17.8)
Total number of individuals	24	23	27	

### Proviral load and mRNA assessment

Total DNA and RNA were extracted from enriched CD4^+^ T cells using a commercial kit (Qiagen GmbH, Hilden Germany) and cDNA was generated using Superscript VILO cDNA synthesis kit (Invitrogen) following the manufacturer's instructions. HTLV-1 proviral load absolute quantification was performed as previously described (Dehee et al., 2002), normalized to human albumin gene. Samples were assayed in duplicate. HTLV-1 proviral load was calculated as follows: copy number of HTLV-1 per 1,000 CD4^+^ T cells = (copy number of HTLV-1)/(copy number of albumin) × 2 × 1,000 cells.

Transcription levels of *Tax* and *HBZ* were measured as previously described (Saito et al., 2009), using housekeeping gene Ubiquitin C (Life Technologies assay ID# Hs00824723_m1) to calculate *tax* and *HBZ* 2^−ΔCt^ relative expression. Relative quantification of 42 RFs using mRNA from CD4^+^ T cells from 13 HTLV-1-infected patients and 5 uninfected healthy controls was performed using custom-made TaqMan® Low Density Arrays previously described (Abdel-Mohsen et al., 2013)(Applied Biosystems, Foster City, CA). RF gene descriptions are given in Table 2.

**Table 2:** List of 42 antiretroviral genes measured in CD4^+^ T cells.

**Target Information**	**NCBI Gene Description**
APOBEC3A-H	Apolipoprotein B mRNA editing enzyme, catalytic polipeptide-like 3
BST2/theterin	Bone Marrow Stromal cell antigen 2
SLFN11	Schlafen family member 11
TRIM5α, TRIM11, TRIM21, TRIM22, TRIM26, TRIM28, TRIM32	Tripartite motif family
CPSF6	Cleavage and Polyadenylation Specific Factor 6
SAMHD1	SAM domain and HD domain 1
PML	Promyelocytic Leukemia protein
RNF114	Ring Finger Protein 114
Trex1	Three Prime Repair Exonuclease 1
RPRD2	Regulation of nuclear Pre-mRNA Domain containing 2
CHFR	Checkpoint with forkhead and ring finger domains, E3 ubiquitin protein ligase
ISG15	ISG15 ubiquitin-like modifier
EIF2AK2/PKR	Eukaryotic translation initiation factor 2-alpha kinase 2
CTR9/PAF1/RTF1	CTR9, PAF1, RTF1/RNA polymerase II complex component
IFITM family (3 members)	Interferon induced transmembrane protein
RSAD2 (viperin)	Radical S-adenosyl methionine domain containing 2
MOV10	Moloney leukemia virus 10, homolog
HERC5	HECT domain and RLD 5
MX2	MX dynamin like GTPase 2
CDKN1A	Cyclin-dependent kinase inhibitor 1A
BRD4	Bromodomain containing 4
RNASEL	Ribonuclease L (2′,5′-oligoisoadenylate synthetase-dependent)
CH25H	Cholesterol 25-Hydroxylase
LGALS3BP	Lectin, Galactoside binding Soluble 3 Binding Protein
CNP	2′,3′-cyclic nucleotide 3′ phosphodiesterase
IFI16	Interferon gamma Inducible protein 16

### Microarray analysis

Total RNA extraction (RNeasy kit, QIAGEN, Venlo, the Netherlands) and Whole Genome microarray analysis (HuGene 1.0 ST array, Affymetrix, Santa Clara, CA) were performed according to the manufacturers' specifications. Data were analyzed using the Bioconductor limma package. Microarray data used in this study are available at Gene Expression Omnibus under GEO accession number GSE82160 (Brazilian HTLV-1 cohort), GSE29333 (UK HTLV-1 cohort, Tattermusch et al.) and GSE18233 (Swiss HIV cohort, Rotger et al., 2010).

### Enrichment and evolutionary analysis

Genome-wide enrichment analysis was performed using a modified Fisher's test, considering the total number of annotated genes for which transcripts were detectable by microarray (*n* = 22,370), followed by stringent FDR (Benjamini–Hochberg) correction. Data and full methodology on human candidate retroviral restriction factors and chimp/human cross-species adaptive evolution and purifying selection during primate evolution are detailed in (Osame, 1990; Singh et al., 2014). In brief, measurements of cross-species adaptive evolution used the McDonald-Kreitman value to compare human sequence to chimpanzee in a set of 15,052 protein-coding genes (Singh et al., 2014) and purifying selection during primate evolution was measured using the proportion of nonsynonymous (*K*_A_) over synonymous (*K*_S_) substitutions per site in five primates (human, chimpanzee, orangutan, rhesus, and common marmoset) in a set of 140 candidate genes selected for their relationship to HIV pathogenesis (Osame, 1990).

### Statistical analysis

Statistical analysis was performed using GraphPad Prism software (GraphPad Software, version 6 and 7, San Diego, CA). Non-parametric statistical tests (Mann–Whitney, Wilcoxon tests, and Spearman correlation) were used for patient data, with Bonferroni correction for multiple comparisons between RFs, PVL and *tax/HBZ* mRNA levels. Transcriptome-wide correlation of mRNA expression levels with either set point viral load (HIV-1) or TRIM5 (HTLV-1) mRNA expression was calculated using Spearman's correlation, followed by FDR correction for multiple testing.

## Results

### Antiretroviral RFs separate in two major clusters, positively and negatively associated with HTLV-1 virological and clinical status

**Figure 1:**
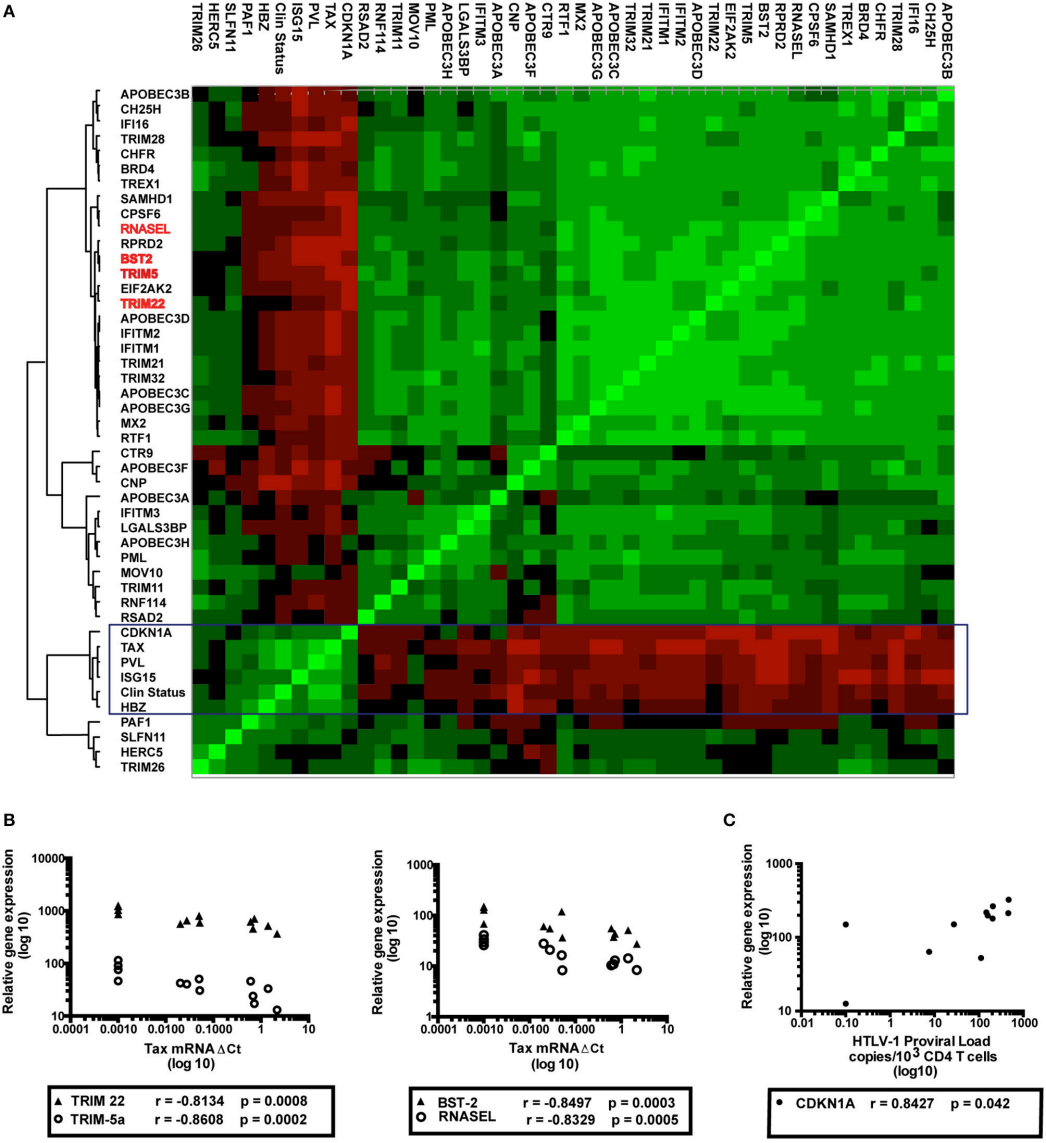
Association between host restriction factors (RFs) expression, HTLV-1 virological parameters and clinical status of HTLV-1 infected individuals. **(A)** Analysis of mRNA levels of 42 RFs, Tax/HBZ and HTLV-1 proviral load (PVL) in CD4^+^ T cells from 6 asymptomatic carriers (AC), 7 HAM/TSP patients and 5 healthy donors (HD). Expression of host RFs was quantified by TLDA, results were clustered according to Spearman's correlation and shown as heatmap (red = negative, green = positive). The boxed cluster indicates a minor subset of “proviral” RFs (CDKN1A, ISG15) clustering with HTLV-1 PVL, *Tax* mRNA, *HBZ* mRNA, and clinical status (HD/AC/HAM), whereas the majority of RFs forms a large “antiviral” cluster. A subset of antiviral RFs (TRIM5α, TRIM22, BST2, and RNASEL) with statistically significant negative correlation to tax mRNA are highlighted in red. **(B)** Correlation between TRIM5α, TRIM22, BST2, RNASEL, and tax mRNA **(C)** Correlation between CDKN1A and tax mRNA in HTLV-1-infected individuals (AC *n* = 6, HAM *n* = 7).

Based on a previously defined subset of host genes with significant anti-HIV activity (Abdel-Mohsen et al., 2013), we used a custom-made array to quantify the transcriptional levels of 42 RFs (listed in Table 2) in peripheral CD4^+^ T cells from HTLV-1 infected patients or healthy controls. We compared the gene expression profiles of 13 HTLV-1-infected patients (6 AC, 7 HAM/TSP) and 5 uninfected subjects using cluster analysis. As evident from Figure 1A, two major RF subsets appeared as separate clusters. First, a minor cluster of RFs (CDKN1A/ISG15 and PAF1) positively correlates with biomarkers of HTLV-1 disease (PVL, mRNA levels of *Tax* and *HBZ*, as well as clinical status, box Figure 1A). Second, the largest cluster of RFs was found to correlate negatively with HTLV-1 biomarkers (Figure 1A). Within this large cluster, negative correlations between the expression levels of Tax and TRIM5α (*r* = −0.86; *p* = 0.0084), TRIM22 (*r* = −0.81; *p* = 0.0336), BST2 (*r* = −0.85; *p* = 0.0126), and RNASEL (*r* = −0.83; *p* = 0.021) were statistically significant after stringent Bonferroni correction (Figure 1B). Conversely, cyclin-dependent kinase inhibitor 1A (CDKN1A, also known as *p21*), a regulator of cell-cycle progression, was positively associated with HTLV-1 proviral load (*r* = 0.84; *p* = 0.042) (Figure 1C). Surprisingly, these *ex vivo* correlations recapitulate the *in vivo* findings of IFN-β treatment in an immunovirological trial in HAM/TSP, in which IFN-β was found to significantly decrease both *tax* mRNA levels and lymphoproliferation (Oh et al., 2005), but not PVL. In contrast, *in vivo* IFN-α treatment of HAM/TSP patients was found to decrease PVL and immune activation (Izumo et al., 1996). Therefore, we investigated if IFN-α/β might differentially affect lymphoproliferation and genome-wide expression profiles in HAM/TSP.

### *Ex vivo* lymphoproliferation is significantly down-regulated by IFN-β, but not IFN-α in HAM/TSP

**Figure 2:**
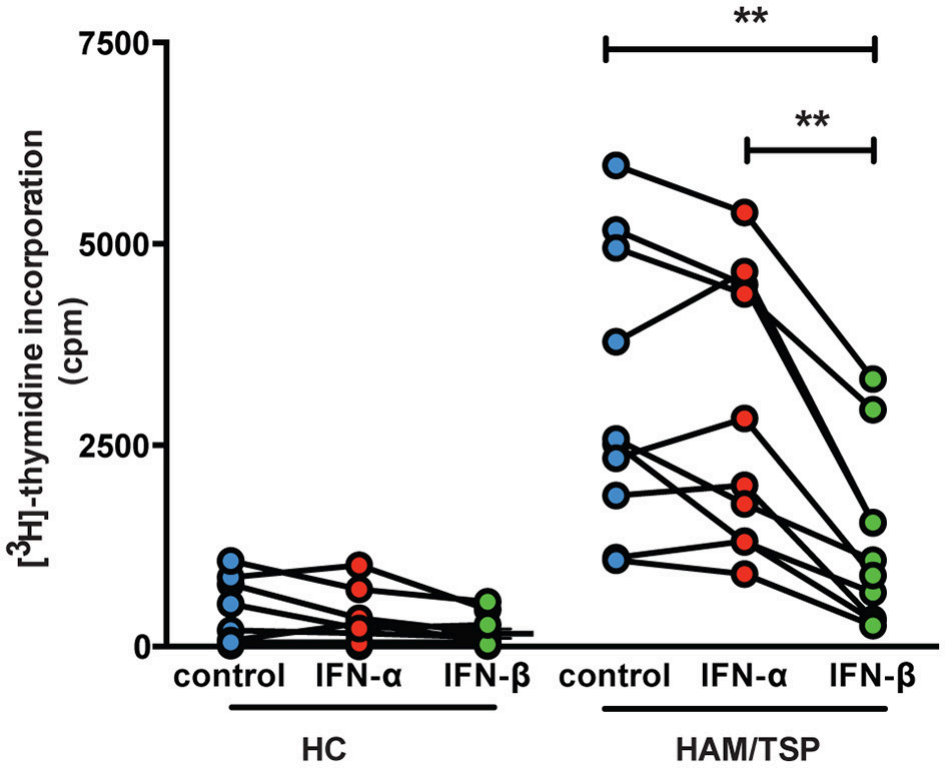
*Ex vivo* lymphoproliferation is significantly down-regulated by IFN-β, but not IFN-α in HAM/TSP. Spontaneous lymphoproliferation was measured by [^3^H]-thymidine incorporation, as previously described Moens et al. (2012a). The effect of IFN-α and IFN-β (1000 IU/ml) upon *ex vivo* lymphoproliferation was determined in healthy controls (HC, *n* = 11) and HAM/TSP patients (*n* = 10). ^**^p < 0.01.

We found that lymphoproliferation was significantly inhibited by IFN-β (*p* < 0.01), but not by IFN-α (Figure 2) This differential effect was not due to a defect in IFN-α bioactivity, since both IFNs displayed equal antiviral activity in a standardized Vesicular Stomatitis Virus (VSV) bioassay (Dierckx et al., 2017). This effect was specific for HAM/TSP, since no significant IFN-induced antiproliferative effect was observed in both healthy controls (Figure 2) and HTLV-1-infected carriers (data not shown).

### Genome-wide analysis identifies selective up-regulation of RFs by IFN-β, but not IFN-α, in HAM/TSP

**Figure 3:**
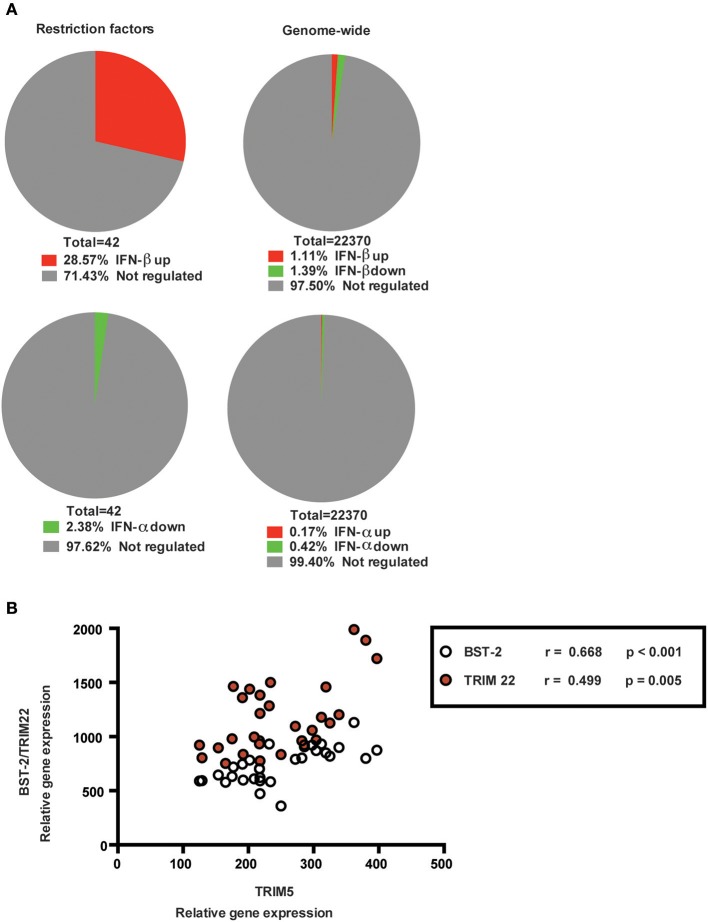
Genome-wide analysis of PBMCs from HAM/TSP patients of RFs after *ex vivo* treatment with type I interferons**. (A)** Microarray analysis to determine genes significantly up- or down-regulated after 48 h *ex vivo* treatment of PBMCs from HAM/TSP patients with 1000 IU/ml IFN-β (*n* = 5), upper right corner pie-chart or IFN-α (*n* = 6), lower right corner pie-chart. RFs were significantly enriched among IFN-β-regulated, (upper left corner, genome-wide enrichment *p* < 0.0001), but not IFN-α-regulated (lower left corner, *p* > 0.05) genes. A total of 22370 genes were analyzed. **(B)** Reanalysis of published *ex vivo* whole blood transcriptomes from an independent HTLV-1 UK cohort (Tattermusch et al., 2012). Correlation between TRIM5α and TRIM22/BST2 in both HTLV-1-infected AC (*n* = 17) and HAM/TSP patients (*n* = 10).

Since RFs can be IFN-regulated, we used microarray analysis to assess whether the proviral and antiviral clusters might be selectively up- or down-regulated by either IFN-α or IFN-β, the two IFN subtypes previously used in HAM/TSP trials (Izumo et al., 1996; Oh et al., 2005). As shown in Figure 3A, genome-wide analysis identified 12/42 RFs (including TRIM5α, TRIM22, and BST2) were significantly up-regulated by IFN-β (*n* = 5, enrichment *p* < 0.0001), vs. 0/42 by IFN-α treatment (*n* = 6, *p* > 0.05) during short-term (48 h) *ex vivo* culture of PBMC from HAM/TSP patients. In a striking parallel to our results in CD4^+^ T cells, where TRIM5 displayed the strongest negative correlation to Tax mRNA, TRIM5 was also identified as the top IFN-β-induced gene among the significant genes (*p* = 0.0009).

### Genome-wide analysis identifies a strongly co-regulated TRIM5α/TRIM22/BST2 subset in HTLV-1 infection, independent of the HAM/TSP disease signature

To validate our findings, we analyzed published *ex vivo* whole blood transcriptomes in an additional HTLV-1-infected cohort (Tattermusch et al.; detailed in Table 1) (Osame et al., 1986). As shown in Figure 3B, significant positive correlations were confirmed between TRIM5α/TRIM22 (*p* = 0.005, *r* = 0.50) and TRIM5α/BST2 (*p* < 0.001, *r* = 0.67) in both HTLV-1-infected AC (*n* = 17) and HAM/TSP patients (*n* = 10). Of note, TRIM5α mRNA expression was not significantly correlated to IFITM1 mRNA levels (data not shown), which is the only RF (among 42 studied herein) that is present in the HAM/TSP disease signature.

### RFs correlate oppositely to *in vivo* retroviral RNA levels in untreated HTLV-1 vs. HIV-1 infection

**Figure 4:**
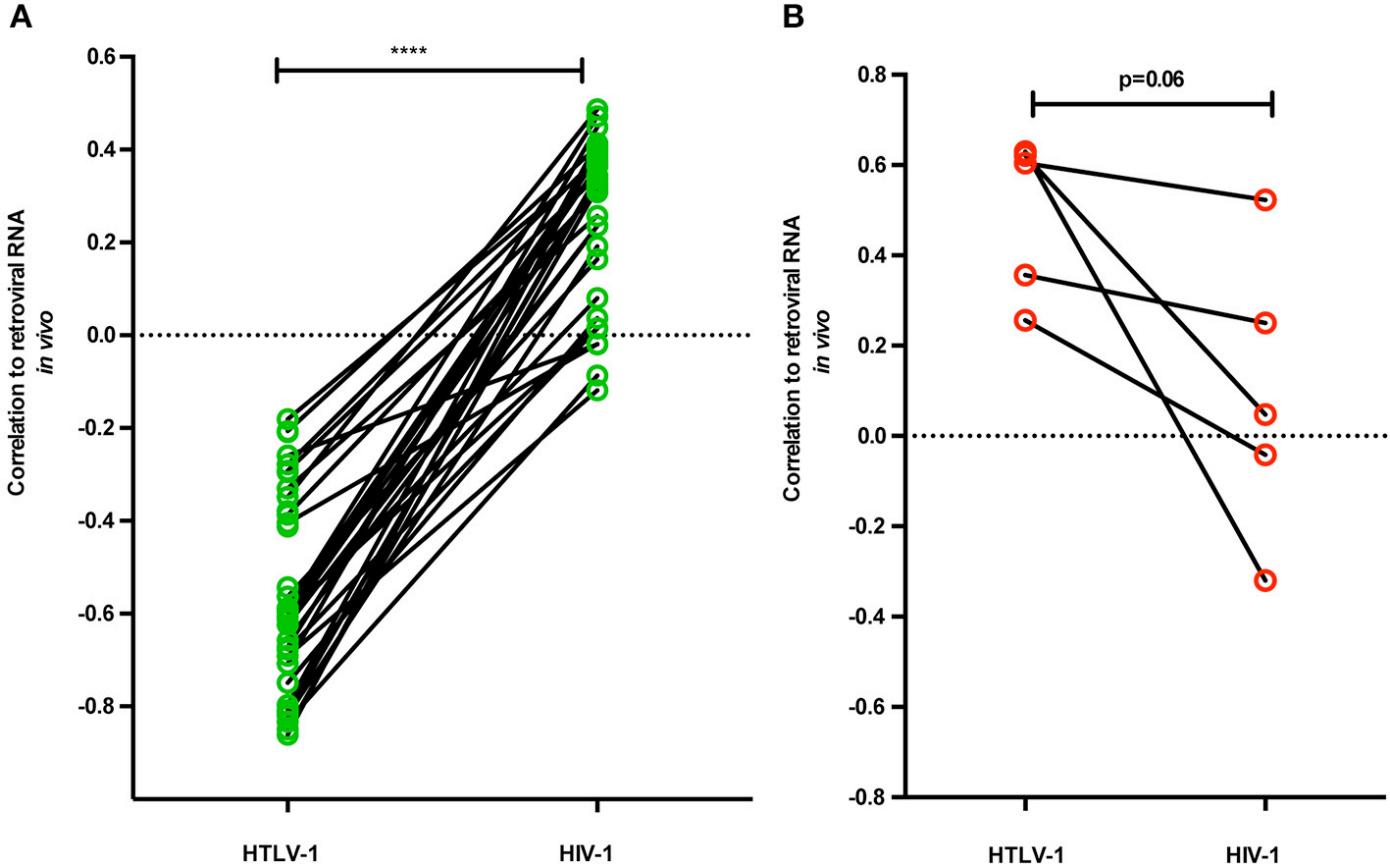
Restriction factors correlate oppositely to *in vivo* retroviral RNA levels in untreated HTLV-1 vs. HIV-1 infection. A pairwise analysis was performed for RFs in antiviral and proviral clusters, by correlating to *in vivo* RNA levels in both retroviral infections, using HTLV-1 data from this study (Figure 1A) and data from the Swiss HIV cohort (Rotger et al., 2010). Out of 42 RFs, 40 were matched to unique transcripts in HIV-1 microarray data, followed by calculating transcriptome-wide correlation of viral load set point to each separate gene, using stringent FDR (Benjamini-Hochberg) correction. **(A)** HTLV-1 “antiviral” cluster genes show significantly increased (positive) correlation to HIV-1 *in vivo* RNA levels (^****^p < 0.0001, Wilcoxon signed rank test, *n* = 35). **(B)** HTLV-1 “proviral” cluster genes show a tendency for decreased (positive) correlation to HIV-1 *in vivo* RNA levels (*p* = 0.06, Wilcoxon signed rank test, *n* = 5).

Surprisingly, the “proviral/deleterious” gene cluster identified by its strong positive correlation to HTLV-1 RNA levels and PVL contains the RFs previously demonstrated to exert a robust protective anti-HIV-1 effect *in vivo* and *in vitro*, namely *CDKN1A, SLFN11, PAF1*, and *ISG15* (Telenti, 2005; Ortiz et al., 2009; McLaren et al., 2015; Nozuma et al., 2017). Therefore, we performed a pairwise analysis of correlation to *in vivo* RNA levels in both retroviral infections, using our HTLV-1 data (Figure 1A) and data from the Swiss HIV cohort (Rotger et al., 2010) by calculating transcriptome-wide correlation of viral load set point to each separate gene, followed by FDR correction. As shown in Figures 4A,B, a striking opposite effect can be observed for the “proviral” vs. “antiviral” clusters. HTLV-1 “antiviral” cluster genes show significantly increased (higher *R*-values) correlation to HIV-1 *in vivo* RNA levels (*p* < 0.0001, Wilcoxon signed rank test, Figure 4A). The reverse phenomenon can be observed for the HTLV-1 “proviral” cluster genes, showing a tendency for decreased (lower *R*-values) correlation to HIV-1 *in vivo* RNA levels (*p* = 0.06, Wilcoxon signed rank test, Figure 4B).

### Lentivirus-driven primate evolution has shaped antiretroviral activity in untreated HTLV-1 infection

**Figure 5:**
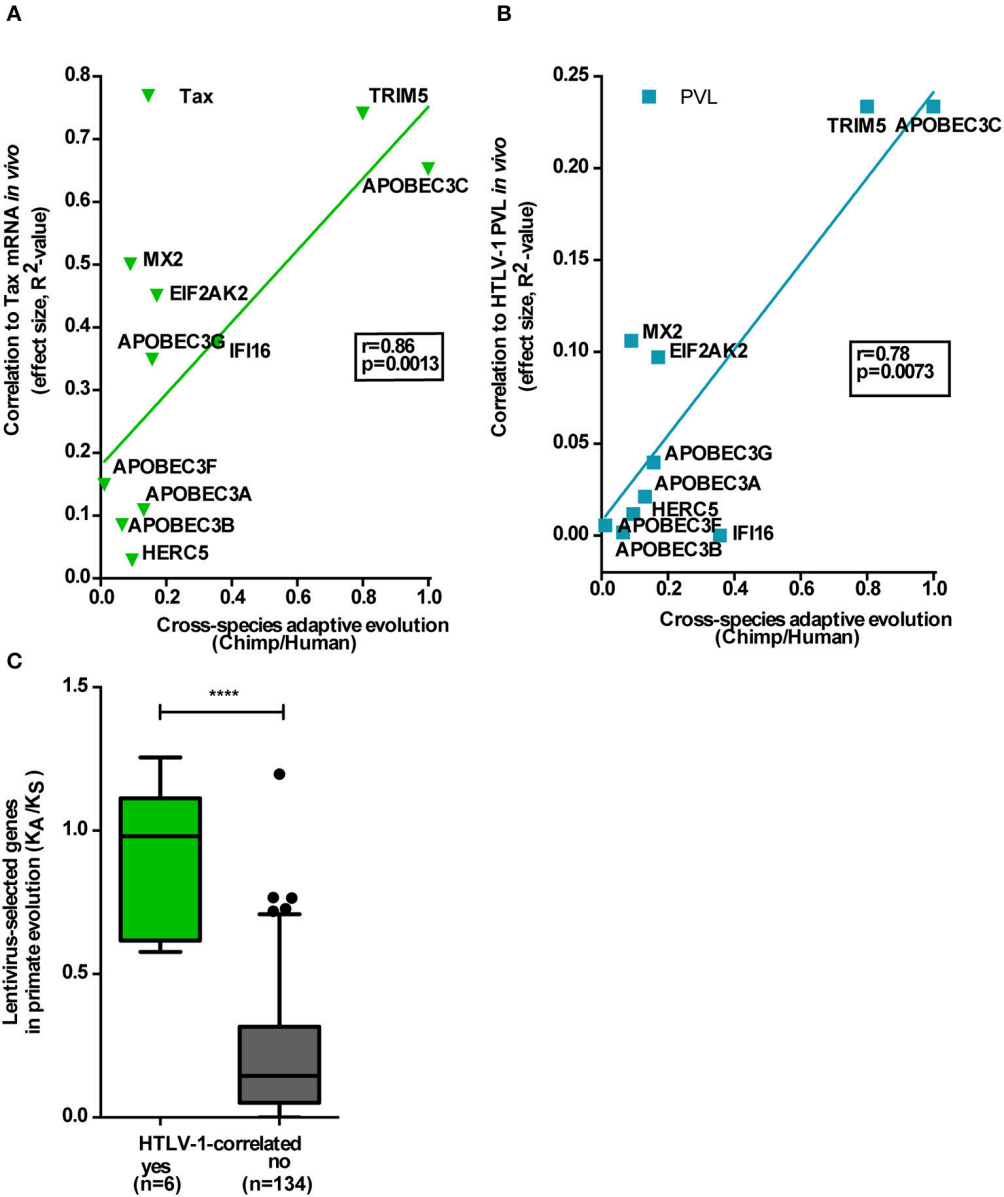
Lentivirus-driven primate evolution has shaped antiretroviral activity in untreated HTLV-1 infection. The antiviral RF cluster is strongly selected during recent primate evolution, as measured by both cross-species adaptive evolution between chimpanzees and humans and purifying selection throughout primate evolution. **(A,B)** Among the 42 anti-HIV RFs selected in this study, 10 were found to display chimpanzee/human cross-species adaptive evolution (McLaren et al., 2015). The anti-HTLV-1 effect (measured as *R*^2^) of these 10 genes was significantly correlated to their adaptive evolution score, for both Tax mRNA (*r* = 0.86, *p* = 0.0013) and PVL (*r* = 0.78, *p* = 0.0073). **(C)** Six genes of the proposed antiviral cluster underwent lentivirus-driven purifying selection (measured as K_A_/K_S_, Ortiz et al., 2009) during primate evolution, which was significantly increased vs. all other lentivirus-selected primate genes (*n* = 134, ^****^p < 0.0001, Mann–Whitney test).

Lentivirus infections, to which HIV and its ancestral SIV belong, have had a pronounced effect upon primate evolution (Telenti, 2005), whereas no similar evidence exists for deltaretroviruses, such as HTLV or PTLV. As shown in Figure 5, the antiviral RF cluster we identified for HTLV-1 is strongly selected during recent primate evolution, as measured by both cross-species adaptive evolution between chimpanzees and humans (Figures 5A,B) and purifying selection throughout primate evolution (Figure 5C). Among the 42 anti-HIV RFs selected in this study, ten (TRIM5, APOBEC3A-B-C-F-G, IFI16, HERC5, EIF2AK2, and MX2) were found to display chimp/human adaptive evolution, as described by McLaren et al. (2015). The anti-HTLV-1 effect (measured as *R*^2^ to represent effect size) of these 10 genes was significantly correlated to their adaptive evolution score, for both Tax mRNA (*r* = 0.86, *p* = 0.0013, Figure 5A) and PVL (*r* = 0.78, *p* = 0.0073, Figure 5B). In agreement with our results in Figures 1A,B, the effect size was greater for Tax mRNA (Figure 5A—range 0.1–0.74) than for PVL (Figure 5B—range 0.0–0.23), underscoring the putative role of these RFs in viral replication, rather than clonal expansion. Only six genes of the proposed “antiviral” cluster (TRIM5, TRIM22, and BST2, as well as APOBEC3F-G-H) underwent lentivirus-driven purifying selection during primate evolution (Ortiz et al., 2009), measured as K_A_/K_S_. In addition, these six HTLV-1-correlated genes underwent a significantly stronger selection during primate evolution, as compared to all other lentivirus-selected primate genes (*n* = 134, *p* < 0.0001, Mann–Whitney test, Figure 5C).

## Discussion

The striking negative association between mRNA levels of *Tax* and RFs TRIM5α, TRIM22, BST2, and RNASEL, despite the small number of participants in the study, suggests that these RFs may represent important mechanisms of defense against HTLV-1 infection and/or neuroinflammation. Our hypothesized antiviral activity of TRIM5 against HTLV-1 is supported by the recent findings of Nozuma et al. demonstrating a significant association between TRIM5 R136Q polymorphism and lower PVL, specific to HAM/TSP patients (Nozuma et al., 2017). Strikingly, polymorphisms in TRIM5, TRIM22, and BST2, but not APOBECs or TREX1 were significantly associated to another neuroinflammatory disorder, multiple sclerosis (Nexo et al., 2013). Conversely, the apparent proviral effect of PAF-1, ISG15, and CDKN1A are in agreement with the recently demonstrated IFN-inducible HAM/TSP disease signature (Tattermusch et al., 2012). Moreover, the robustness of the antiviral and proviral clusters we identified in this study indicates they represent plausible candidate biomarkers for future HAM/TSP clinical trials. In agreement with Saito et al., who demonstrated a positive correlation between HBZ mRNA levels and HAM/TSP disease severity (Saito et al., 2009), our cluster analysis shows the strongest association between HBZ and clinical status (Figure 1A). However, no RFs were significantly correlated to HBZ mRNA levels after correction for multiple testing, in contrast to tax mRNA levels (Figure 1B). This might be due to post-transcriptional regulation of HBZ mRNA vs. protein levels, as Baratella et al. recently demonstrated cytoplasmatic HBZ protein as a biomarker able to discriminate between AC and HAM/TSP (Baratella et al., 2017). Due to their significant association with all currently used surrogate markers (PVL, *Tax*, and *HBZ* mRNAs), as well as clinical status, antiviral and proviral clusters are likely to reflect positive vs. negative therapeutic outcomes, respectively. Therefore, our results help explain the “IFN paradox” in HAM/TSP, by reconciling apparently conflicting data in the literature, namely the existence of an interferon-inducible disease signature and the demonstrated beneficial therapeutic effects of type I interferons, both IFN-α and β in HAM/TSP (Izumo et al., 1996; Oh et al., 2005).

In addition, our study also reveals striking differences between these two widely used IFN subtypes. First, a significant antiproliferative effect of β and not α was observed in HAM/TSP patients, corroborating our recent findings in ATL (Dierckx et al., 2017) and revealing a surprising similarity between these two highly distinct HTLV-1-associated pathologies. On the other hand, our study also reveals another parallel between neuroinflammatory disorders HAM/TSP and multiple sclerosis, namely their unique sensitivity to IFN-β over IFN-α. Of note, our results provide mechanistic evidence for the previously described immunovirological and clinical impact observed *in vivo* with IFN-β therapy in HAM/TSP patients, in an open-label trial (Oh et al., 2005), as well as two case reports with remarkable clinical response (Costa et al., 2012; Viana et al., 2014). This superior antiproliferative effect of IFN-β in HAM/TSP, as compared to IFN-α, parallels our findings in the other major HTLV-1-associated disease, Adult T-cell Leukemia (Dierckx et al., 2017).

Surprisingly, the “proviral/deleterious” gene cluster, identified by its strong positive correlation to HTLV-1 RNA levels and PVL, contains the RFs previously demonstrated to exert the strongest protective anti-HIV-1 effect *in vivo* and *in vitro*. Thus, p21 (*CDKN1A*), schlafen 11 (*SLFN11*), and PAF1 were strongly associated with reduced CD4+ T cell-associated HIV RNA during antiretroviral treatment (Abdel-Mohsen et al., 2015). *SLFN11* was also identified as overexpressed in HIV-positive “elite controllers” (Abdel-Mohsen et al., 2013), who maintain undetectable viral load, even in the absence of antiretroviral treatment. PAF1 was identified by a genome-wide screen for HIV RFs (Liu et al., 2011) and restricts HIV-1, HIV-2, and SIV *in vitro*. Finally, CDKN1A was demonstrated as a robust antiviral effect against HIV-1 replication *in vitro*, by different groups (Leng et al., 2014; Farberov et al., 2015).

With up to eight percent of the human genome of retroviral origin, the importance of retroviruses for the evolution of susceptible host organisms cannot be overestimated (Ortiz et al., 2009; Lascano et al., 2015). Over millions of years, Lentivirus infections (retroviruses including HIV and ancestral SIV) have had a pronounced effect upon primate evolution (Ortiz et al., 2009). Yet, no evidence exists for deltaretroviruses (including HTLV and PTLV). Using two different quantitative approaches, i.e., measuring chimp/human cross-species adaptive evolution (Figures 5A,B) and purifying selection throughout primate evolution (Figure 5C), we found that the antiviral RF cluster we identified for HTLV-1 was under strong purifying selection during recent primate evolution. Our preselection of a small number of candidate RFs, by virtue of their proven antiviral effect against HIV-1, is an obvious limitation of the first part of our study, focusing on purified CD4+ cells. However, we confirm the unique TRIM5/TRIM22/BST2 antiviral cluster by two independent unbiased methods, i.e., PBMC transcriptomics and genome-wide evolutionary analysis of protein-coding genes. By comparing the only two pathogenic human retroviruses, we found striking differences in *in vivo* correlations of “candidate” RFs to retroviral RNA levels, as an indirect measure of their possible anti-HIV and anti-HTLV-1 activity. Although some antiviral effector molecules such as cGAS exhibit “panviral” activity (Schoggins et al., 2014), our results argue against “panretroviral” restriction factors in the human setting. Very few host genes, such as CIITA and PKR (also known as EIF2AK2), have been formally demonstrated to restrict both HIV-1 and HTLV-1 replication *in vitro* (Schoggins et al., 2011; Tosi et al., 2011; Cachat et al., 2013; Kinpara et al., 2013; Forlani et al., 2016). Interestingly, CIITA interacts and cooperates with TRIM22 in restricting replication of HIV-1 (Forlani et al., 2017). However, CIITA is unique among RFs because it is transcriptionally upregulated by IFN-gamma but downregulated by IFN-β (Lu et al., 1995). In contrast, IFN-α strongly upregulates PKR/EIF2AK2 in HTLV-1-infected cells but does not decrease Tax or Hbz mRNA (Moens et al., 2012b; Cachat et al., 2013; Kinpara et al., 2013). Thus, CIITA and PKR are unlikely candidates for the IFN-β-mediated antiviral and antiproliferative effect we observed *ex vivo* and *in vitro* in HAM/TSP patients. Unfortunately, high-throughput *in vitro* analysis of RFs, such as performed by Schoggins et al. for HIV-1 and several other viruses, are lacking for HTLV-1 (Schoggins et al., 2011). Based on our previous demonstration of a 1000-fold difference in HIV-1 vs. HTLV-1 antiviral effect in co-infected MT-4 cells (Moens et al., 2012b) we anticipate strong differences might be observed for *in vitro* HTLV-1 vs. HIV-1 restriction pathways. As a final layer of complexity, HIV-1 also displays striking discrepancies between *in vivo* and *ex vivo* RF antiviral activity (Rotger et al., 2010; Schoggins et al., 2014). Therefore, we believe the term “restriction factors” should be used with caution in human retrovirology. “Antiviral effector genes” might be a more precise term, if defined within a precise context, i.e., depending on cell type, virus and upstream regulators such as IFN subtypes. Regarding cell type-specificity, TRIM5α and TRIM22 expression at both mRNA and protein level has been shown to predominate in CD4^+^ T cells, as compared to other mononuclear cell types (Singh et al., 2014), which explains the strong agreement between our RF analysis in CD4^+^ T cells and IFN response in PBMCs, as well as whole blood analysis from UK cohort participants (Tattermusch et al., 2012).

Mechanistically, both the antiviral vs. “proviral” effects of TRIM5, CDKN1A, and ISG15 might also be explained by broader cellular processes, outside the narrow definition of RFs. TRIM5 is an intracellular protein that exerts its protective effect by disrupting the retroviral capsid as it transports viral nucleic acid into the nucleus. In addition, innate immune signaling might contribute to TRIM5-mediated restriction. Lascano et al. have shown that activation of innate immune signaling is conserved among primate and carnivore TRIM5 orthologs and that such activity is required for TRIM5-mediated restriction activity (Lascano et al., 2015). CDKN1A upregulation by Tax might shorten G1 phase by promoting formation of stable kinase complexes, contributing to cell-cycle progression (Kehn et al., 2004) and proliferation of infected clones. Regarding ISG15, our group has recently demonstrated its anti-inflammatory extracellular cytokine-like activity, through monocyte-specific induction of IL-10 (Dos Santos et al., 2018). However, recent data reveal an unexpected IL-10-induced proliferative switch in HAM/TSP-derived HTLV-1-infected T-cell lines (Sawada et al., 2017). Therefore, ISG15 inducing IL-10 and hence triggering lymphoproliferation might represent, at least in part, a molecular mechanism for the putative “proviral” role of ISG15 in HAM/TSP. Integrating the data from Tattermusch et al. (2012), Dos Santos et al. (2018), Sawada et al. (2017) and this study, we hypothesize that an “IFN-beta like/TRIM5” gene signature vs. a “non-IFN-beta like/ISG15” gene signature might be predictive of HAM/TSP disease progression, as well as of therapeutic outcome with immunomodulatory and/or antiproliferative drugs (IFNs, glucocorticoids and others).

In conclusion, our integrated *ex vivo* approach reveals that antiretroviral genes in HTLV-1 infection and HAM/TSP cluster in two distinct “proviral/antiviral” classes, of which the TRIM5α/TRIM22/BST2 antiviral subset, selected during recent primate evolution, is selectively up-regulated by IFN-β signaling in HAM/TSP. Our results thus provide *ex vivo* mechanistic evidence for the observed *in vivo* immunovirological effect of IFN-β treatment in HAM/TSP and identify biomarkers as well as possible therapeutic targets for a precision medicine approach. Finally, a protective antiviral and IFN-inducible TRIM5α/TRIM22/BST2 gene cluster, independent of the HAM/TSP IFN-inducible disease signature, reconciles the apparent IFN paradox in HTLV-1 research and confirms type I IFN as a two-edged sword in human health and disease.

## Author contributions

FL, RR, and JVW: conceived and designed the experiments; SM, FL, EC, PB, and JVW: performed the experiments; FL, SM, LG, RR, RK, JV, TD, and JVW: analyzed the data; EK, DN, AS, BG-C, and LG: contributed reagents, materials, and analysis tools; FL, SM, JVW, and DN: wrote the paper.

### Conflict of interest statement

The authors declare that the research was conducted in the absence of any commercial or financial relationships that could be construed as a potential conflict of interest.
